# Polysubstance Use in Early Adulthood: Patterns and Developmental Precursors in an Urban Cohort

**DOI:** 10.3389/fnbeh.2021.797473

**Published:** 2022-01-27

**Authors:** Annekatrin Steinhoff, Laura Bechtiger, Denis Ribeaud, Manuel P. Eisner, Boris B. Quednow, Lilly Shanahan

**Affiliations:** ^1^Jacobs Center for Productive Youth Development, University of Zurich, Zurich, Switzerland; ^2^Institute of Criminology, University of Cambridge, Cambridge, United Kingdom; ^3^Experimental and Clinical Pharmacopsychology, Department of Psychiatry, Psychotherapy and Psychosomatics, Psychiatric Hospital of the University of Zurich, Zurich, Switzerland; ^4^Neuroscience Center Zurich, University of Zurich and Swiss Federal Institute of Technology, Zurich, Switzerland; ^5^Department of Psychology, University of Zurich, Zurich, Switzerland

**Keywords:** substance use, polysubstance use, early adulthood, risk factors, longitudinal, community, latent class

## Abstract

Polysubstance use (i.e., simultaneous or sequential use of different psychoactive substances) is associated with increases in the risk of severe health problems and social impairments. The present study leverages community-representative, long-term longitudinal data from an urban cohort to assess: (a) the prevalence and continuation of polysubstance use between adolescence and early adulthood; (b) different patterns of polysubstance use (i.e., combinations of substances) in early adulthood; and (c) childhood risk factors for polysubstance use in early adulthood. At age 20 (*n* = 1,180), respondents provided comprehensive self-reported information on past-year substance use, including use of legal and illicit substances (e.g., cannabinoids, stimulants, and hallucinogens), and nonmedical use of prescription drugs (e.g., opioids, tranquilizers). In adolescence (ages 13–17), limited versions of this questionnaire were administered. In childhood (ages 7–11), potential risk factors, including individual-level factors (e.g., sensation-seeking, low self-control, aggression, and internalizing symptoms) and social-environmental factors (e.g., social stressors, exposure to others’ substance use), were assessed. We fitted latent class models to identify classes of participants with different substance use profiles in early adulthood. The results show that polysubstance use increased between early adolescence and early adulthood. The continuation of polysubstance use was common (stability between all adjacent assessments: odds ratio >7). At age 20, more than one-third of participants reported polysubstance use (involving illicit substances, nonmedical use of prescription drugs, and cannabidiol). Four latent classes with polysubstance use were identified: (1) broad spectrum of substances; (2) cannabis and club drugs; (3) cannabis and the nonmedical use of prescription drugs; and (4) different cannabinoids. Risk factors for any polysubstance use included childhood sensation-seeking and exposure to others’ substance use; some childhood risk factors were differentially associated with the four classes (e.g., low self-control in childhood was associated with an increased likelihood of being in the broad spectrum class). The classes also differed with regard to socio-demographic factors. This study revealed that polysubstance use is a widespread and multifaceted phenomenon that typically emerges during adolescence. To facilitate the design of tailored prevention mechanisms, the heterogeneity of polysubstance use and respective socio-demographic and developmental precursors need to be considered.

## Introduction

The use of psychoactive substances (e.g., cannabinoids, hallucinogens, stimulants, opioids, including their nonmedical use) is a threat to young people’s health (United Nations, 2018). Risks associated with substance use include physical, psychological, social, and functional impairments. These are multiplied when individuals consume two or more psychoactive substances simultaneously or sequentially, for example during the previous year (i.e., polysubstance use; World Health Organization, 1994). Indeed, compared to the use of a single substance, polysubstance use is associated with more dangerous patterns of substance use (e.g., addiction, overdose), physical health problems (e.g., injury), premature mortality, comorbid risk-taking (e.g., violence, dangerous driving), self-harming behaviors (e.g., suicidal behaviors), psychopathology (e.g., depressive symptoms), cognitive dysfunctions (e.g., impaired executive functions and empathy), and poorer educational and occupational achievements (European Monitoring Centre for Drugs and Drug Addiction, 2002; Conway et al., 2013; Connor et al., 2014; Kroll et al., 2018; Crummy et al., 2020). Encountering these consequences of polysubstance use during early adulthood could be especially harmful, as young people are expected to master important transitions in their educational, professional, social, and identity development (Arnett, 2000).

Community-based research on the developmental course of polysubstance use during adolescence and early adulthood is scarce. This is due, in part, to the lack of assessment of substances other than alcohol, tobacco, and cannabis before late adolescence in previous work (Connor et al., 2014). The* first aim of our study* was to examine the prevalence and stability of polysubstance use (defined here as the use of at least two psychoactive substances during the previous year) between early adolescence and early adulthood. We leveraged data from a representative, urban community sample (Ribeaud and Eisner, 2010; Shanahan et al., 2020; Ribeaud et al., 2021) with prospective longitudinal assessments from childhood to early adulthood, and substance use assessments beginning in early adolescence.

The different patterns of polysubstance use are also poorly understood. Research has begun to use person-centered analysis, including latent class analysis (LCA), to learn more (for reviews, see Connor et al., 2014; Tomczyk et al., 2016). These studies typically identified a group with no use, groups with limited to medium range use (e.g., use of a single substance, such as alcohol, or additional use of select other substances, especially cannabis), and a broad range group. The latter typically subsumed all users of illicit substances and those engaged in the non-medical use of prescription drugs (Connor et al., 2014). This is not surprising, given that the numbers of individuals using illicit substances were often too low in these studies to disaggregate this group further, or illicit substance use was assessed with summary items not differentiating between particular substances in the first place (Tomczyk et al., 2016; Carbonneau et al., 2021). Our *second aim* was to better understand heterogeneity within the polysubstance use group, which can only be done based on samples with high rates of substance use assessed with comprehensive substance use questionnaires, as is the case here (Quednow et al., 2021).

The most cost-effective approach to lowering the burden from polysubstance use would be to prevent and intervene before adolescents begin to engage in this pattern of use. Yet, we have a limited understanding of the childhood risk factors that predict (different patterns of) polysubstance use. Based on cross-sectional, retrospective, or short-term longitudinal data, prior research has identified individual-level (e.g., sensation-seeking, psychopathology) and social-environmental (e.g., exposure to others’ substance use, including in family and peer contexts) correlates of polysubstance use (Russell et al., 2015; Tomczyk et al., 2016; Tan et al., 2020; Carbonneau et al., 2021; Crane et al., 2021). The literature on childhood risk factors for *any* adolescent and early adulthood substance use also suggests putative predictors, including additional individual-level (e.g., self-control, risk-taking and externalizing behaviors), and social factors (e.g., social stress; e.g., Wills and Stoolmiller, 2002; Chapple et al., 2005; Barrett and Turner, 2006; Kelly et al., 2015). The *third aim* of our study was to identify childhood risk factors (i.e., precursors; Murray et al., 2009) of any polysubstance use and its different patterns in young adulthood.

Polysubstance use likely reflects different motivations for and instrumentalizations of substance use (Müller and Schumann, 2011; Valente et al., 2020). These include, for example, curiosity, craving social connectedness (Ter Bogt and Engels, 2005), enhancing one’s energy or ability to focus, calming and relaxing (LeClair et al., 2015), and enhancing or counteracting the (side) effects and withdrawal symptoms of other substances (Boys et al., 2001; Winstock et al., 2001; Licht et al., 2012). We assumed that some of these motivations may already be reflected in specific childhood precursors. Therefore, we hypothesized that different childhood individual-level (e.g., internalizing symptoms, sensation-seeking, risk-taking behaviors and delinquency) and social environmental factors (e.g., exposure to others’ substance use, social stressors) predict polysubstance use and its different patterns in young adulthood. For example, the inclination to experiment with new experiences and to take risks (e.g., indicated by sensation-seeking, offensive, and risky behaviors) and a low inhibition threshold (e.g., indicated by low self-control) could signal risk of any later polysubstance use and of a broad range of substances used in particular. In turn, childhood social stressors (e.g., victimization experiences) or internalizing symptoms could signal the risk of nonmedical use of prescription drugs for self-medication purposes. Finally, when children are exposed to others’ substance use, they may assume that substance use is common and safe. Thus, exposure to other’s substance use could precede polysubstance use in general. We examine all of these potential associations by making use of the long-term longitudinal design of our data.

## Materials and Methods

### Participants and Procedures

The data came from the longitudinal Zurich Project on the Social Development from Childhood to Adulthood (*z-proso*; Ribeaud and Eisner, 2010; Eisner et al., 2019; Ribeaud et al., 2021). Participants were selected using a cluster-stratified randomized sampling approach. In 2004, a sample of 1,675 children from 56 primary schools was randomly selected from 90 public schools in the city of Zurich, Switzerland’s largest city. Stratification was performed by considering the school sizes and socioeconomic backgrounds of the school districts. The sample was largely representative of first-graders attending public school in the city of Zurich. Participants were assessed eight times between 2004 (age 7) and 2018 (age 20).

The current study uses data collected at ages 13 (*n* = 1,365), 15 (*n* = 1,446), 17 (*n* = 1,306), and 20 (*n* = 1,180), to examine the developmental course of polysubstance use over time. To investigate different patterns of polysubstance use in early adulthood, and the associated developmental precursors (assessed before age 13), we included those who participated at age 20. Of these participants, 51% were male. Consistent with Switzerland’s immigration policies and the city’s diverse population, participants’ parents were born in over 80 different countries. The majority of participants were born in Switzerland (90%). The educational background of their parents was diverse; in 30% of households, at least one parent held a university degree. The mean (M) household occupational status, measured using the International Socio-Economic Index of Occupational Status (ISEI; Ganzeboom et al., 1992), was 47.1 [standard deviation (SD) = 19.7]. This internationally comparable index of socio-economic status was based on occupation-specific income and the required educational level with scores ranging from 16 (e.g., unskilled worker) to 90 (e.g., judge).

This study is consistent with national and international ethical standards and was approved by the relevant ethics committee. Adolescents provided written consent for their study participation, and parents of those aged 15 and younger could decline their child’s participation in the study. Data were collected from groups of 5–25 participants in classroom settings with paper-and-pencil questionnaires up to age 17 and in a computer laboratory setting with computer-administered surveys at age 20. Completing the surveys typically took about 90 minutes. Adolescents received a cash incentive for their participation, which increased from approximately $30 at age 13 to $75 at age 20.

### Sample Attrition

In z-proso, the highest participation rate was reached at age 15 (*n* = 1,446). Of those who participated at age 15, females were more likely than males to participate again at age 20 (84.5% vs. 76.9%, *p* < 0.001). Those with at least one parent holding a university degree were more likely to participate at age 20 than those whose parents held a lower educational degree (95.0% vs. 79.4%, *p* < 0.001), and those with at least one Swiss-born parent were more likely to participate than those whose parents were both born abroad (83.9% vs. 77.9%, *p* = 0.004). Those who responded at age 20 had a higher adolescent family socio-economic status than those who had dropped out of the survey [ISEI score: *M* = 47.1 (SD = 19.7) vs. *M* = 40.4 (SD = 16.6), *p* < 0.001]. Further details on attrition can be found elsewhere (Eisner et al., 2019; Quednow et al., 2021). Such attrition patterns are common in long-term longitudinal research (e.g., Gustavson et al., 2012; Sigurdson et al., 2014; Steinhoff and Keller, 2020). Our handling of missing data is described in the “Analytic Strategy” section.

### Variables

#### Substance Use at Age 20

Participants were asked how often they had used the following substances during the previous 12 months (exempting any use of medical drugs that were prescribed by a physician): (1) tobacco (e.g., cigarettes, shisha/hookah); (2) beer, wine, alcopops; (3) liquor (e.g., vodka, whisky, gin); (4) cannabinoids, including cannabis (e.g., hashish, grass, weed, marijuana, cannabis), cannabidiol (CBD; e.g., CBD-enriched hemp, cigarettes with CBD-enriched hemp, CBD tinctures), synthetic cannabinoids (i.e., cannabis substitutes such as “Dutch Orange,” “Spice,” “K2,” “Ganja Style”); (5) stimulants, including cocaine, and amphetamine/methamphetamine (e.g., “Speed”, “Pepp”, “Ice”, “Crystal Meth”); (6) empathogenes such as MDMA and its analogues (“Ecstasy,” “Molly”); (7) hallucinogens, including LSD/psilocybin (e.g., “Magic Mushrooms,” “Truffles”), 2C substances (e.g., “Bromo,” “Erox,” “Nexus,” “Venus”), and ketamine (“Special K,” “Vitamin K”); (8) opioids, including heroin and the nonmedical use of codeine-based cough medicine and opioid painkillers; (9) the nonmedical use of benzodiazepine tranquilizers; and (10) anabolic steroids. Assessments were made on a six-point scale (1 = never, 2 = once, 3 = two to five times, 4 = monthly, 5 = weekly, and 6 = daily).

#### Substance Use Between Ages 13 and 17

During adolescence, a limited range of substances was assessed. The list of substances was gradually expanded over time (age 13: alcohol, tobacco, and cannabis; ages 15 and 17: alcohol, tobacco, cannabis, MDMA, cocaine, amphetamine/methamphetamine, and LSD/psilocybin). Assessments of the frequency of use during the previous year were made on the same six-point scale that was used at age 20.

#### Coding of Polysubstance Use

We created dummy variables indicating whether participants had used specific substances at least once during the previous year. A sum score was computed, counting the number of different substances used. This score was then dichotomized to indicate any polysubstance use (i.e., at least two different substances used) vs. no polysubstance use (i.e., single or no substance use) for the analysis of the prevalence of polysubstance use. Furthermore, individuals were assigned to groups with polysubstance use, single substance use, and no use for the analysis of respective developmental precursors. For descriptive comparisons of the prevalence of polysubstance use over time, three different operationalizations of polysubstance use were applied. First, to compare the prevalence of polysubstance use between ages 13 and 20, we included alcohol, tobacco, and cannabis only because these were assessed during all assessments. Second, we computed an indicator of polysubstance use based on all five illicit substances assessed at ages 15, 17, and 20, excluding alcohol and tobacco. Third, an indicator of polysubstance use at age 20 included all substances (excluding alcohol and tobacco) that were assessed using the extended comprehensive questionnaire, which was administered for the first time at that age. This score mainly represents illicit substance use and non-medical use of prescription drugs. Although several CBD products are freely available in Switzerland, we included them in this score as well, because the effects of CBD are different from those of Δ^9^-tetrahydrocannabinol (THC), which is typically more dominant in cannabis (Freeman et al., 2019), and thus, the motivations underlying the use of CBD products vs. cannabis are likely also different (e.g., to relax vs. get high). Indeed, CBD itself is mildly psychoactive with sedative and anxiolytic effects, at least at moderate doses (Bergamaschi et al., 2011; Zuardi et al., 2017). To adjust the aggregate score of polysubstance use for potential overlap of using CBD products and cannabis, an additional sensitivity analysis of the prevalence of polysubstance use at age 20 was conducted, with CBD products being excluded.

For the identification of different polysubstance use patterns at age 20, the dummy variables of the different substances, excluding alcohol and tobacco, were used in subsequent LCAs (see the section on “Analytic Strategy”). For alcohol and tobacco, we created additional dummy variables indicating frequent alcohol and tobacco use (i.e., categories 5 = weekly and 6 = daily were coded 1, and less frequent or no use was coded 0). These were used as potential correlates of latent class membership (see the “Results” section for the underlying rationale).

#### Childhood Risk Factors

First, we included psychological factors and indicators of children’s functioning. All factors were self-reported at age 11, except sensation-seeking, which was a behavioral measure assessed at age 7. The descriptive statistics reported here refer to the age-20 participants.

(a) *Sensation-seeking*: behavioral measure based on an adapted nine-item version of the Travel Game from Alsaker and Gutzwiller-Helfenfinfer (2010), see also Murray et al. (2020a,b), who reported an Omega reliability of 0.80; using a cardboard game, children’s preference for sensational vs. less sensational situations was assessed [e.g., “you must decide whether you want to travel by fast motorbike or funny steam locomotive” (0 = sensational situation not chosen, 1 = sensational situation chosen)]; a sum score was used; recoded scale 0–1 (*M* = 0.57, SD = 0.25);
(b) *Low self-control*: 10 items (e.g., “I often act on the spur of the moment without stopping to think”) from Grasmick et al. (1993), Cronbach’s *α* = 0.75, scale 1 = fully untrue to 4 = fully true (*M* = 1.94, SD = 0.46);
(c) *Aggression*: 15 items from the physical, proactive, indirect, reactive, and oppositional aggression subscales of the Social Behavior Questionnaire by Tremblay et al. (1991), *α* = 0.82; participants were asked to indicate how often during the previous 6 months they had engaged in particular aggressive behaviors (e.g., “physically attacked other people”); scale from 1 = never to 5 = very often (*M* = 1.48, SD = 0.37);
(d) *Internalizing symptoms*: eight items from Tremblay et al. (1991); participants were asked how often during the previous month they had particular feelings (e.g., “I was scared, ” “I was sad without knowing why”); *α* = 0.79, scale from 1 = never to 5 = very often (*M* = 2.06, SD = 0.65);
(e) *Childhood onset of any substance use*: three items asked about any previous use of alcohol, tobacco, and cannabis at age 11, which were combined and dichotomized to indicate any substance use—this scale differed from that used in subsequent substance use assessments (9% of the sample reported substance use at age 11);
(f) *Risky media use*: three items assessed whether the participants had ever watched adult horror movies, adult action movies, or played adult computer games (e.g., “have you ever watched 18+ rated horror movies, that is to say, movies only meant for adults”; yes/no)—items were combined to indicate any use of adult media (42% of the sample reported risky media use at age 11);
(g) *Delinquency*: nine items assessed particular behaviors during the previous year (e.g., “stolen something from a shop or kiosk that is worth more than 50 CHF” [yes/no]), and a sum score was created (*M* = 0.97, SD = 1.19).

Second, we included social-environmental factors, which were also self-reported at age 11, unless otherwise indicated:

(a) *Harsh parenting*: five items from the Alabama Parenting Questionnaire (Shelton et al., 1996) indicating parents’ response when the child “misbehaves” or is “disobedient” (e.g., “do your parents spank you with their hand”, with scores from 1 = never to 4 = always) were combined and children with scores in the top quartile of the sample were assigned 1 = harsh parenting and compared to 0 = no harsh parenting (Shanahan et al., 2021), 21% of the sample were assigned 1;
(b) *Bullying victimization*: four items from the Zurich Brief Bullying Scale (Murray et al., 2021); participants were asked to indicate how often in the previous 12 months others had, for example, “ignored or excluded you” or “laughed at, mocked, or insulted you”; *α* = 0.72, scale 1 = never to 6 = almost every day (*M* = 1.81, SD = 0.79);
(c) *Exposure to friends’ substance use*: participants named their two best friends and reported whether these had used alcohol, tobacco, or other substances (e.g., cannabis) during the previous year [i.e., three items for each friend (yes/no)]; we created a binary variable indicating whether at least one friend had used any substances; 9% of the sample reported friends’ substance use at age 11;
(d) *Maternal substance use during pregnancy*: mother-report, provided in the first assessment wave; three binary items assessing any use of alcohol, tobacco, and any other substances during pregnancy (yes/no)—items were combined to indicate 1 = any substance use during pregnancy vs. 0 = no use (37% of the sample had been exposed to maternal substance use).

#### Socio-demographics

We included children’s *sex* (0 = female, 1 = male), *socio-economic background* assessed as ISEI (Ganzeboom et al., 1992), and *parental migration background* (0 = at least one Swiss born parent, 1 = both parents born abroad).

### Analytic Strategy

First, we calculated and compared the prevalence of any polysubstance use between early adolescence (age 13) and early adulthood (age 20) in an effort to identify typical developmental periods of onset. The maintenance of polysubstance use over time was examined by testing the stability of polysubstance use between adjacent assessments, using binary logistic regression models (i.e., polysubstance use at one assessment was regressed on polysubstance use at the respective previous assessment).

Second, we applied LCA, to identify clusters of participants with different patterns of polysubstance use in early adulthood. In this step of the analysis, we included all participants who reported the use of at least two substances other than alcohol and tobacco at age 20. Our decision on the optimal number of classes was based on relative fit indices [Bayesian Information Criterion (BIC) and Akaike’s Information Criterion (AIC)] and entropy as an indicator of classification precision. Lower BIC and AIC were considered indicative of better model fit; entropy >0.80 and class counts >5% were considered indicative of model accuracy (Muthen, 2004). Furthermore, solutions with varying numbers of classes were inspected graphically for conceptual interpretability (Masyn, 2013). Finally, we tested associations between childhood precursors and individuals’ polysubstance use status (i.e., any polysubstance use vs. no use and single substance use) and their most likely class membership using nominal logistic regression analyses.

In the LCA, missing data was accounted for by applying full information maximum likelihood estimation; in the regression models, we used multiple imputations to handle missing data on predictor variables (Schafer and Graham, 2002; Enders, 2013). For each model that we present, all variables included in that model were involved in the imputation model, and 20 imputed data sets were generated. Parameter estimates were averaged across the imputed data sets. Based on these procedures, we were able to include all age-20 participants (*n* = 1,180) in the analyses of the precursors of polysubstance use status and all age-20 participants with any polysubstance use (*n* = 420) in the analyses of the precursors of polysubstance use patterns. Descriptive analyses and tests of stability over time were conducted using SPSS V25; all other analyses were conducted using Mplus V8.

## Results

### Prevalence and Stability of Polysubstance Use Between Early Adolescence and Early Adulthood

**Figure 1:**
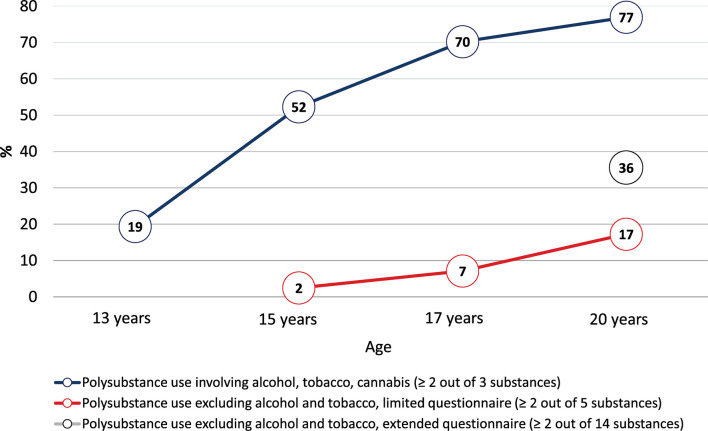
Prevalence of polysubstance use between ages 13 and 20 years.* Note*. The prevalence of polysubstance use at age 20 was 25% when cannabidiol (CBD) was excluded.

The prevalence of past-year polysubstance use increased between early adolescence and early adulthood (Figure 1). According to our *first operationalization (i.e., alcohol, tobacco, cannabis)*, the increase was especially sharp between ages 13, when one in five adolescents had used at least two of the three substances in the previous year, and 17, when more than two-thirds of adolescents had used at least two of the three. At age 20, more than three-quarters of early adults reported past-year polysubstance use.

According to our *second operationalization (i.e., substances other than alcohol and tobacco, using the limited questionnaire administered starting in mid-adolescence)*, past-year polysubstance use became increasingly prevalent during late adolescence. At age 17, 1 in 14 adolescents reported illicit polysubstance use; this number increased to one in six at age 20. Importantly, this latter number was more than doubled when using the *third operationalization (i.e., extended, comprehensive questionnaire used at age 20 only)*. Specifically, almost one in three early adults reported polysubstance use according to this operationalization. While the previous numbers are useful for comparison purposes, this latter number, being based on the comprehensive questionnaire, is the most reliable reflection of the true polysubstance use prevalence in our sample. Therefore, our comparative descriptive analyses show that prevalence rates based on narrow assessments of only a small selection of different substances are likely to severely underestimate the prevalence of polysubstance use in a population.

A sensitivity analysis excluding CBD from the age-20 polysubstance use score revealed a polysubstance use prevalence of 25%, reflecting that use of CBD largely overlapped with the use of cannabis (97% of those reporting the use of CBD products also reported cannabis use; 46% of those reporting cannabis use also reported the use of CBD products).

**Figure 2:**
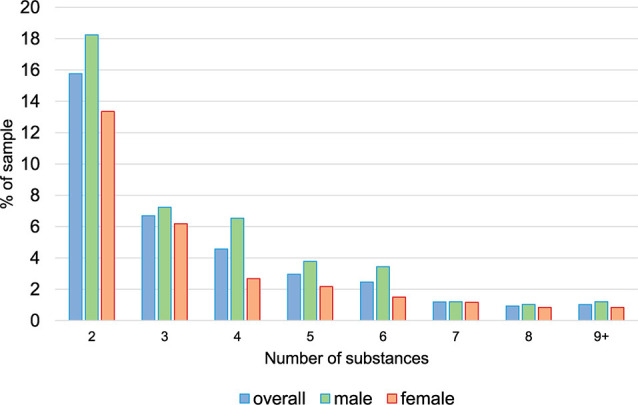
Prevalence of counts of substances (based on extended questionnaire and excluding alcohol and tobacco) used during the previous year at age 20. *Note*. Categories nine and higher were combined due to low prevalence.

At age 20, the number of different substances used ranged from 0 to 13 (Figure 2), and the average number of substances used among those with any polysubstance use (excluding alcohol and tobacco) was 3.53 (SD = 1.98). This count did not differ between males and females with any polysubstance use (*p* = 0.381).

The stability of polysubstance use over time was high, including when the alcohol-tobacco-cannabis coding was used [from age 13 to age 15: odds ratio (OR) = 7.55, 95% confidence interval (CI) = 5.22–10.92; from 15 to 17: OR = 13.52, 95% CI = 9.77–18.71; from 17 to 20: OR = 16.66, 95% CI = 11.87–23.38] and when the narrow coding involving illicit substances and excluding alcohol and tobacco was used (age 15–17: OR = 23.89, 95% CI = 11.35–50.30; age 17–20: OR = 14.78, 95% CI = 8.78–24.88). These findings show that, once initiated, polysubstance use is often continued over time.

### Patterns of Polysubstance Use in Early Adulthood

Our investigation of different patterns of polysubstance use in early adulthood focused on illicit substance use, legal drugs other than alcohol and tobacco (in Switzerland, this includes CBD), and the nonmedical use of prescription drugs. We excluded any past-year use of alcohol and tobacco because the latter two were so prevalent in our sample (Quednow et al., 2021) that they would not differ much among the latent classes. However, after identifying the different latent classes, we also examined the prevalence of frequent (i.e., weekly or daily) consumption of alcohol and tobacco within these classes.

**Figure 3:**
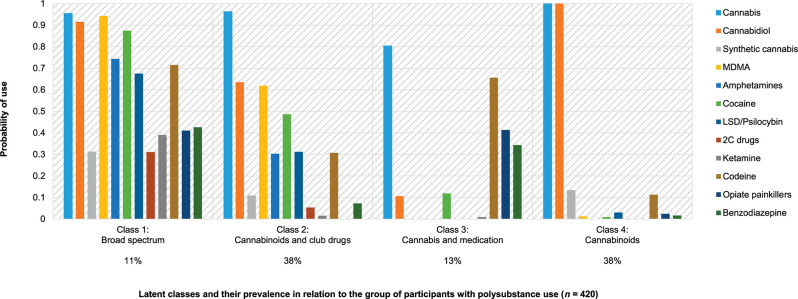
Polysubstance use profiles at age 20. *Note*. Prevalence based on estimated model.

The LCA included all participants who reported the use of at least two substances other than alcohol and tobacco during the previous year (36% of the sample, *n* = 420). Heroin and anabolic steroids were excluded from this analysis due to their very low prevalence (*n* < 5). Based on a comparison of relative fit indices (Table 1), a three-, a four-, and a five-class solution were selected for further inspection of interpretability and class sizes. The four-class solution, with the lowest BIC, revealed distinct classes with substantially different substance use profiles (see Figure 3) and reasonable prevalence (>10% each). Because this met our criteria for the best solution, we chose the four-class solution for further analysis.

**Table 1:** Model fit and precision of latent class solutions with one to five classes.

Number of classes	BIC	AIC	Entropy
1	4,410.72	4,362.24	–
2	4,099.42	3,998.41	0.81
3	4,032.29	3,878.76	0.82
*4*	*4,015.96*	*3,809.90*	*0.82*
5	4,054.75	3,796.17	0.83

*Note. Italics indicate the solution chosen for further analyses. BIC, Bayesian Information Criterion; AIC, Akaike’s Information Criterion*.

The following four classes were identified (for the prevalence of each class, see Figure 3):

(1) Class 1, “*broad spectrum*,” included participants who used many substances (e.g., cannabinoids, stimulants, hallucinogens, and opioids)—although this was the smallest class, more than one in 10 early adults with polysubstance use belonged to it;
(2) Class 2, “*cannabinoids and* *club drugs*, ” was characterized by the use of fewer substances than class 1, involving cannabinoids plus stimulants, empathogenes, and hallucinogens that are typically consumed in party contexts, and, in some cases, also codeine—one-third of early adults with polysubstance use belonged to this class,
(3) Class 3, “*cannabis and medication*, ” was also characterized by medium-range substance use but primarily involved cannabinoid use plus nonmedical use of prescription drugs, including those with opioid and tranquilizers—one in eight early adults with polysubstance use belonged to this class;
(4) Class 4, “*cannabinoids*, ” was primarily characterized by the use of different cannabinoids. Together with class 2, this class comprised the largest group: more than one-third of the sample with polysubstance use was in it.

To further characterize the classes, we investigated their association with weekly or daily alcohol and tobacco use. The likelihood of drinking alcohol frequently was higher among classes 1 [64%, standard error (SE) = 8.2], 2 (61%, SE = 4.4), and 4 (47%, SE = 4.2) than among class 3 (10%, SE = 5.4). The comparisons between class 3 and all other classes were significant (*p* < 0.001). The difference between classes 2 and 4 was also significant (*p* = 0.022). Smoking tobacco frequently was more prevalent among members of classes 1 (81%, SE = 6.9) and 2 (69%, SE = 4.2) compared to those of classes 3 (39%, SE = 7.3; *p* < 0.001 and *p* = 0.001 for comparison with classes 1 and 2, respectively) and 4 (50%, SE = 4.2; *p* < 0.001, *p* = 0.002, respectively). Altogether, these findings show that the spectrum of substances used by members of classes 1 and 2 was supplemented by both frequent alcohol and tobacco use, whereas substances used by classes 3 and 4 were more selective.

### Risk Factors for Polysubstance Use

First, we investigated the socio-demographic and childhood precursors of any polysubstance use compared to single and no substance use at age 20. Notably, the majority of individuals reporting single substance use reported cannabis use (*n* = 259), and only a minority (*n* = 40) reported the use of another substance as their only drug of choice (excluding alcohol and tobacco). We specified separate models for each precursor, adjusting for socio-demographics, and a multivariable model including all precursors in one model (i.e., a “full model”).

The results revealed precursors of polysubstance use from all three domains: (1) socio-demographics; (2) individual-level factors (i.e., psychological factors and indicators of functioning); and (3) social-environmental factors (Table 2). Although male participants were more likely than females to report polysubstance use rather than single or no substance use, the sex difference was not significant when individual and social-environmental factors were included in the model simultaneously (i.e., the full model). A higher socio-economic background was associated with an increased risk of substance use (i.e., poly- or single use compared to no use). Children with two Swiss parents had a higher risk than those with a parental migration background to engage in polysubstance use compared to single or no substance use. In addition, childhood-onset of risk-taking behaviors, such as substance use and risky media use, was associated with an increased risk of substance use (i.e., single or poly, respectively) in early adulthood compared to no use. Childhood sensation-seeking and exposure to maternal substance use during pregnancy were also associated with an increased risk of polysubstance use compared to no use. Substance use by friends during childhood was uniquely associated with a higher risk of later polysubstance use compared to single substance use.

**Table 2:** Associations between potential risk factors and early adulthood substance use status (nominal logistic regressions: OR, 95% CI).

Precursors	Poly- vs. single substance use	Poly- vs. no substance use	Single vs. no substance use
**Associations adjusted for socio-demographics**		
*Socio-demographics*		
Socio-economic background	1.00 (0.99–1.01)	**1.01** (1.00–1.02)	**1.01** (1.00–1.02)
Migration background	**0.61** (0.44–0.86)	**0.51** (0.37–0.69)	0.83 (0.60–1.15)
Male sex	**1.77** (1.31–2.40)	**1.92** (1.46–2.52)	1.08 (0.81–1.46)
*Childhood individual factors*			
Sensation-seeking	1.78 (0.89–3.56)	**3.94** (2.05–7.58)	**2.22** (1.11–4.44)
Low self-control	1.46 (0.99–2.16)	**2.28** (2.58–3.29)	**1.57** (1.04–2.37)
Aggression	1.28 (0.81–2.02)	**2.07** (1.34–3.21)	1.62 (0.97–2.70)
Internalizing symptoms	1.00 (0.77–1.30)	1.14 (0.90–1.45)	1.14 (0.87–1.49)
Substance use	1.35 (0.89–2.04)	**2.24** (1.54–3.27)	**1.67** (1.10–2.53)
Risky media use	**1.53** (1.10–2.14)	**2.14** (1.56–2.92)	**1.40** (1.00–1.95)
Delinquency	**1.20** (1.04–1.39)	**1.34** (1.15–1.57)	1.12 (0.94–1.34)
*Social environmental*			
Harsh parenting	1.22 (0.86–1.73)	**1.58** (1.14–2.20)	1.30 (0.92–1.85)
Bullying victimization	1.07 (0.87–1.32)	1.18 (0.97–1.44)	1.10 (0.87–1.40)
Friends’ substance use	**1.53** (1.02–2.31)	**1.85** (1.27–2.68)	1.21 (0.80–1.84)
Maternal substance use pregnancy	1.35 (0.99–1.84)	**1.57** (1.18–2.09)	1.16 (0.85–1.58)
**Full model (including all predictors)**		
*Socio-demographics*			
Socio-economic background	1.00 (0.99–1.01)	**1.01** (1.00–1.02)	**1.01** (1.01–1.02)
Migration background	**0.60** (0.42–0.86)	**0.47** (0.33–0.66)	0.77 (0.55–1.01)
Male sex	1.29 (0.89–1.87)	1.10 (0.79–1.54)	0.87 (0.60–1.24)
*Childhood individual factors*			
Sensation-seeking	1.41 (0.68—2.90)	**2.74** (1.41–5.34)	1.90 (0.94–3.86)
Low self-control	1.07 (0.67–1.72)	1.48 (0.96–2.29)	1.35 (0.83–2.19)
Aggression	0.90 (0.50–1.62)	1.14 (0.66–1.95)	1.25 (0.66–2.34)
Internalizing symptoms	0.89 (0.67–1.18)	0.94 (0.72–1.23)	1.07 (0.81–1.42)
Substance use	0.94 (0.60–1.48)	1.51 (0.99–2.32)	**1.64** (1.05–2.56)
Risky media use	1.37 (0.94–1.99)	**1.58** (1.13–2.22)	1.18 (0.82–1.68)
Delinquency	1.11 (0.95–1.30)	1.11 (0.95–1.31)	1.00 (0.84–1.20)
*Social environmental factors*			
Harsh parenting	1.21 (0.78–1.90)	1.17 (0.79–1.76)	0.99 (0.63–1.57)
Bullying victimization	1.02 (0.82–1.29)	1.07 (0.84–1.35)	1.03 (0.81–1.32)
Friends’ substance use	**2.15** (1.01–4.61)	1.18 (0.62–2.22)	0.53 (0.24–1.19)
Maternal substance use pregnancy	1.35 (0.98–1.88)	**1.55** (1.13–2.12)	1.15 (0.83–1.59)

*Note. Bold print indicates significant associations at *p* < 0.05*.

Comparisons between the four polysubstance use classes were based on regression models that included one childhood risk factor at a time and adjusted for socio-demographics. The rationale for testing one predictor at a time only was that the smallest class count was *n* = 44, meaning that full models with all predictors were not feasible. The results revealed differences between class members in socio-demographic and individual-level factors (Table 3). Low self-control characterized the members of the broad-spectrum class (class 1) compared to all other classes. Early adults in class 1 were also characterized by higher levels of childhood delinquency compared to those reporting the use of cannabinoids and club drugs (class 2) or the use of cannabinoids only (class 4). Early adults with a parental migration background were more likely to be in the cannabis and medication class (class 3) than in any other class. Females were also more likely to be in the cannabis and medication class (class 3) compared to the cannabinoids and club drugs (class 2) and cannabinoids (class 4) classes but not compared to the broad spectrum class (class 1). Members of the cannabinoids class (class 4) had lower levels of childhood aggression than those of the broad spectrum (class 1) and cannabinoids and club drugs (class 2) classes.

**Table 3:** Associations between potential risk factors and latent class membership, adjusted for socio-demographics (nominal logistic regressions: OR, 95% CI).

	Cannabinoids and club drugs (2) vs. Broad spectrum (1)	Cannabis and medication (3) vs. Broad spectrum (1)	Cannabinoids (4) vs. Broad spectrum (1)	Cannabinoids and club drugs (2) vs. Cannabis and medication (3)	Cannabinoids and club drugs (2) vs. Cannabinoids (4)	Cannabis and medication (3) vs. Cannabinoids (4)
*Socio-demographics*	
Socio-economic background	1.01 (0.99–1.03)	1.00 (0.98–1.03)	1.02 (0.98–1.04)	1.01 (0.99–1.03)	0.99 (0.98–1.01)	0.98 (0.97–1.00)
Migration background	0.81 (0.35–1.87)	**3.15** (1.21–8.21)	1.57 (0.69–3.56)	**0.25** (0.12–0.51)	**0.51** (0.31–0.85)	**2.02** (1.01–4.05)
Male sex	1.30 (0.65–2.58)	0.48 (0.21–1.07)	1.20 (0.60–2.37)	**2.72** (1.45–5.13)	1.09 (0.69–1.72)	**0.40** (0.22–0.74)
*Childhood individual factors*						
Sensation-seeking	0.41 (0.07–2.46)	0.18 (0.03–1.30)	0.37 (0.06–2.12)	2.60 (0.63–10.73)	1.15 (0.40–3.36)	0.49 (0.13–1.93)
Low self-control	**0.46** (0.22–0.96)	**0.36** (0.15–0.90)	**0.31** (0.15–0.65)	1.66 (0.73–3.80)	1.58 (0.92–2.70)	1.07 (0.49–2.34)
Aggression	0.70 (0.32–1.54)	0.82 (0.28–2.36)	**0.28** (0.12–0.65)	1.06 (0.37–3.05)	**2.63** (1.36–5.08)	2.56 (0.92–7.13)
Internalizing symptoms	0.84 (0.44–1.59)	1.03 (0.50–2.11)	0.90 (0.47–1.76)	0.79 (0.45–1.38)	0.91 (0.60–1.37)	1.11 (0.63–1.96)
Substance use	0.76 (0.30–1.94)	0.58 (0.21–1.60)	0.51 (0.20–1.28)	1.31 (0.60–2.84)	1.50 (0.81–2.78)	1.14 (0.53–2.43)
Risky media use	1.11 (0.52–2.37)	0.96 (0.39–2.38)	0.76 (0.35–1.66)	1.16 (0.58–2.33)	1.46 (0.89–2.40)	1.26 (0.62–2.56)
Delinquency	**0.76** (0.60–0.96)	0.78 (0.56–1.09)	**0.65** (0.51–0.83)	1.07 (0.76–1.50)	1.18 (0.97–1.44)	1.14 (0.82–1.57)
*Social environmental factors*						
Harsh parenting	1.43 (0.60–3.43)	1.93 (0.73–5.15)	1.19 (0.50–2.83)	0.73 (0.36–1.47)	1.21 (0.72–2.03)	1.64 (0.83–3.25)
Bullying victimization	0.96 (0.62–1.47)	0.90 (0.53–1.53)	0.75 (0.48–1.17)	1.11 (0.70–1.78)	1.29 (0.95–1.76)	1.17 (0.74–1.85)
Friends’ substance use	0.68 (0.29–1.61)	0.51 (0.19–1.38)	0.50 (0.21–1.20)	1.35 (0.62–2.98)	1.38 (0.76–2.50)	1.03 (0.47–2.26)
Maternal substance use pregnancy	0.95 (0.48–1.91)	1.33 (0.57–3.14)	1.02 (0.51–2.04)	0.73 (0.37–1.41)	0.94 (0.60–1.47)	1.31 (0.68–2.50)

*Note. Bold print indicates significant associations at *p* < 0.05*.

## Discussion

Our investigation revealed that polysubstance use increases between early adolescence and early adulthood and is often sustained over time. In our urban community sample with high levels of substance use, the range of different substances used simultaneously or consecutively during the previous year was wider, and patterns of substances combined were more heterogeneous than most prior research had suggested. Several socio-demographic factors and childhood precursors signal individuals’ risk of polysubstance use and differentiate, in part, among diverse patterns of polysubstance use reported in early adulthood.

Our data show that polysubstance use is highly prevalent in young people from this urban community. Even when alcohol and tobacco were excluded from the analyses, polysubstance use as defined here was identified in more than one-third of early adults in our sample when CBD was included; and in one out of four young adults when CBD was excluded. Indeed, the prevalence of any substance use is high in urban Switzerland compared to international evidence (Quednow et al., 2021; Shanahan et al., 2021). This might, in part, be a consequence of relatively high drug availability. However, our comparisons of the prevalence rates based on different operationalizations of polysubstance use, using limited and more extensive questionnaires, indicate that prevalence rates from other studies that assessed only a few different substances likely substantially underestimate the prevalence of polysubstance use in youth. To assess the true prevalence of polysubstance use in a community, it is imperative to administer comprehensive lists of substances available on the local market and to consider the potential nonmedical use of prescription drugs.

Our prospective longitudinal study design with substance use assessments that started in early adolescence provides novel evidence of adolescence as an onset period of polysubstance use. At ages 15 and 17, many adolescents may be experimenting with different substances and use them only once. However, the increasing prevalence of polysubstance use over time and the high odds of polysubstance use continuation indicate that the initiation of polysubstance use in adolescence is a major risk factor for prolonged polysubstance use and perhaps also for the progression to increasingly risky patterns of use (Trenz et al., 2012; Olthuis et al., 2013). Like a vicious cycle, the high and increasing prevalence of polysubstance use in mid- and late-adolescence can, in and of itself, also be a risk factor for (more) individuals to engage in polysubstance use, because exposure to peers’ polysubstance use could increase peer pressure and misjudgments about the dangers associated with polysubstance use (Willis et al., 2019). Indeed, our findings show that exposure to peers’ substance use is a unique developmental precursor of one’s own subsequent engagement in polysubstance use.

Altogether, these findings underscore the importance of awareness and health education campaigns and tailored polysubstance use prevention programs targeting youth *before* they reach mid-adolescence. Given the high prevalence and potentially severe consequences of chronic polysubstance use, parents and professionals (e.g., pediatricians) should also be made aware of these issues. Our results show that childhood sensation-seeking, childhood onset of risk-taking behaviors, and premature exposure to others’ substance use were uniquely associated with an increased risk of early adulthood polysubstance use. Therefore, all these factors represent promising targets or markers for the need of early prevention mechanisms tailored to counteract early adulthood polysubstance use in general.

Notably, the group of early adults engaging in polysubstance use was heterogeneous, both in terms of the combinations of substances used and some of the associated risk factors. All groups were characterized by the consumption of cannabis, but they differed in the additional substances used, which is a common finding in similar research (Quek et al., 2013; Connor et al., 2014). Specifically, the use of club drugs and the use of different cannabinoids (especially cannabis and CBD products) were the most prevalent patterns of polysubstance use in our Zurich-based sample. However, the numbers of early adults reporting nonmedical use of prescription drugs in addition to cannabis use, or the use of a broad range of all different kinds of substances, were also considerable. These insights extend the international evidence on latent classes of polysubstance use by adding Swiss data, which had previously been missing (Connor et al., 2014; Tomczyk et al., 2016).

The different polysubstance use classes may reflect different contexts of and motivations for substance use (Valente et al., 2020). For example, using a broad spectrum of substances (class 1) is likely associated with a motivation to experiment with new experiences (including substance use) and a lifestyle that comes with frequent opportunities to try different substances (e.g., in nightlife contexts). The use of cannabinoids plus medical drugs with opioids (class 3) may represent an attempt to self-medicate among some individuals, to ease pain or to calm down and raise one’s mood for those suffering from anxiety or depressive symptoms (Blume et al., 2000; Shehnaz et al., 2014). However, other individuals in class 3 may simply resort to medication instead of using illicit substances because they perceive medical drugs as less harmful, dangerous, and illegal or because these substances are more easily accessible to them.

Our findings of the associations between risk factors and the different classes show that prevention programs may need to address groups of children with different challenges in different ways. For example, the consistent association between low childhood self-control and membership in the broad spectrum class may reflect these individuals’ low inhibition threshold when facing opportunities to try new substances. Thus, those on track towards experimentation with a broad spectrum of substances may need support in channeling low self-control into less risky behaviors. This prevention may not be particularly relevant for those on track toward other polysubstance use patterns.

However, other childhood factors were not differentially associated with particular polysubstance use patterns. For example, the classes did not differ in terms of childhood internalizing symptoms, although prior research based on cross-sectional data found associations between depressive symptoms and a latent polysubstance use class involving, among others, the use of medication to get high (Conway et al., 2013). Together, ours and prior evidence suggest that differences between the classes in the realm of internalizing symptoms, if any, might have developed only during adolescence and early adulthood.

In fact, differences between the cannabis and medication class (class 3) and other classes mainly pertained to socio-demographic factors in our study. Previous research has found that the female gender is associated with a higher likelihood of self-medication (Shehnaz et al., 2014), and indeed, females with polysubstance use in our sample were relatively likely to be in class 3. In addition, parental migration background increased the likelihood of being in the medication class compared to select other classes. Together, these findings indicate that early adults who represent the typically less privileged members of society (especially females and those with migrant backgrounds) tend to engage in more covert substance use than the more privileged groups (especially males and those with native Swiss parents) who tend to engage in more overt substance use indicated by the broad spectrum (class 1) and club drugs (class 2) classes.

Importantly, the time frame we used to define polysubstance use (i.e., the previous year) is common in polysubstance use research (Conway et al., 2013; Connor et al., 2014) but does not allow us to distinguish between individuals who take different substances sequentially (e.g., to counteract other substance effects or, more independently, based on different motivations and at different occasions) vs. those who consume them simultaneously (e.g., to enhance specific substance effects). However, previous research has shown that the simultaneous use of two or more substances is common among early adults with any past-year polysubstance use, and simultaneous use of alcohol or cannabis with other substances is especially frequent (Quek et al., 2013). Indeed, our follow-up analyses of associations between class membership and alcohol and tobacco use indicated that in some of the classes, frequent (i.e., weekly or daily) use of alcohol and tobacco was common. In turn, weekly or even daily consumption of alcohol or tobacco necessarily implies that any other (combinations of) substances must have been used shortly before or after drinking and smoking or at the same time.

Our study has some limitations. First, substance use was self-reported and could have been underestimated due to social desirability. However, the high rates of substance use reported here suggest that underreporting was not a serious issue. Second, although our list of potential childhood precursors of polysubstance use was comprehensive, some potentially relevant factors were not assessed, such as childhood trauma (Martinotti et al., 2009; Tonmyr et al., 2010; Armour et al., 2014; Davis et al., 2021). Third, although the sample was largely representative of young people growing up in the city of Zurich, it is unclear whether the findings are generalizable to the entire Swiss or international populations. It is likely that some of the substance use characteristics in our sample are Zurich-specific (e.g., high prevalence of cocaine, codeine, and CBD), but that the predictors for polysubstance use are generalizable. Finally, our definition of polysubstance use does not distinguish young people who tried each substance only once during the previous year from those who used different substances regularly. Limiting the concept of polysubstance use to regular use of different substances would likely result in a lower prevalence of polysubstance use, and perhaps also different associations between polysubstance use and specific risk factors. More refined assessments of polysubstance use including information on the frequency of use are needed to better understand different polysubstance use patterns, their prevalence, and precursors.

Our study also had important strengths for advancing scientific knowledge about polysubstance use patterns and their developmental precursors. These include a largely representative, prospective longitudinal study design, a high-resolution assessment of early adulthood substance use, and a sample characterized by a high overall prevalence of substance use. The latter facilitated the detection of several patterns of polysubstance use based on advanced statistical methods and their specific developmental correlates. Notably, prior research with adolescents and early adults often identified one polysubstance use class and compared it to single or no substance use classes (Tomczyk et al., 2016; Choi et al., 2018). Our population with high rates of substance use, our consideration of the nonmedical use of prescription drugs, and our use of LCA on only those who exhibited polysubstance use may have contributed to the identification of multiple distinct polysubstance use groups in our sample. Findings regarding different associations among childhood precursors with these classes illustrate that such nuanced insights into polysubstance use patterns are important for developing tailored prevention programs.

In summary, our investigation provides evidence that polysubstance use is a multifaceted phenomenon that affects a considerable proportion of early adults from the community, and this, in part, follows developmental processes that begin in childhood. To adequately assess polysubstance use and its development across an individual’s life span, future studies need to implement comprehensive assessments of the various substances available on local markets. The heterogeneity of polysubstance use and the specific socio-demographic and developmental factors associated with different patterns of substances used need to be more carefully considered in future research to facilitate the design of tailored prevention mechanisms and curb the individual and social burden that often follows polysubstance use.

## Data Availability Statement

The datasets presented in this article are not readily available because they include sensitive personal information. Requests from scientists for re-analysis can be directed to the first author. Requests to access the datasets should be directed to steinhoff@jacobscenter.uzh.ch.

## Ethics Statement

The studies involving human participants were reviewed and approved by the responsible ethics committee at the Faculty of Arts and Social Sciences, University of Zurich. Participants provided written informed consent at each wave; parents of those aged 15 and younger could choose not to have their child participate in the study.

## Author Contributions

AS, BBQ, and LS conceptualized the manuscript. MPE and DR conducted the z-proso study. LS and BBQ designed the age-20 substance use questionnaire. AS, LB, DR, and MPE coded the data. AS analyzed the data and drafted the manuscript with input from LS, BBQ, and LB. All authors critically revised the manuscript and contributed important intellectual content. All authors contributed to the article and approved the submitted version.

## Conflict of Interest

The authors declare that the research was conducted in the absence of any commercial or financial relationships that could be construed as a potential conflict of interest.

## Publisher’s Note

All claims expressed in this article are solely those of the authors and do not necessarily represent those of their affiliated organizations, or those of the publisher, the editors and the reviewers. Any product that may be evaluated in this article, or claim that may be made by its manufacturer, is not guaranteed or endorsed by the publisher.
